# Transhumeral Amputation with Biceps Tenodesis Sling for Flail Shoulder After Irreversible Brachial Plexus Injury in the Setting of Bionic Reconstruction

**DOI:** 10.3390/jcm15135284

**Published:** 2026-07-06

**Authors:** Alexander Gardetto, Gianluca Marcaccini, Ludovico Coldebella, Reinhold Perkmann, Marco Damiano Pipitone, Warren M. Rozen, Ishith Seth, Roberto Cuomo

**Affiliations:** 1 Competence Center for Bionic Prosthetics, Department of Plastic, Aesthetic and Reconstructive Surgery with Hand Surgery, Brixsana Private Clinic, 39042 Bressanone, Italy; 2 Clinic of Plastic, Reconstructive and Aesthetic Surgery, Padova University Hospital, 35128 Padova, Italy; 3 Department of Plastic and Reconstructive Surgery, University of Siena, 53100 Siena, Italy; ludovico.gss@gmail.com (L.C.); roberto.cuomo@unisi.it (R.C.); 4 Department of Vascular and Thoracic Surgery, Regional Hospital, 39100 Bolzano, Italy; reinhold.perkmann@sabes.it (R.P.); marcodamiano.pipitone@sabes.it (M.D.P.); 5 Department of Plastic and Reconstructive Surgery, Peninsula Health, Frankston, VIC 3199, Australia; warrenrozen@hotmail.com (W.M.R.); ishithseth1@gmail.com (I.S.)

**Keywords:** brachial plexus injury, transhumeral amputation, flail shoulder, biceps tenodesis sling, phantom limb pain, prosthetic integration

## Abstract

**Background**: Irreversible brachial plexus injury can leave patients with a painful, insensate, and nonfunctional flail limb, often after failed reconstructive attempts and with limited remaining surgical options. This study describes a modified transhumeral amputation technique incorporating a passive biceps brachii traction sling to improve residual limb stability and prosthetic readiness in this challenging setting. **Methods**: This retrospective, uncontrolled five-case series assessed the feasibility, perioperative safety, descriptive clinical outcomes, and early prosthetic integration of transhumeral amputation with a biceps brachii-based traction sling. Eligible patients had irreversible brachial plexus palsy, complete loss of useful upper-limb function, chronic shoulder instability or traction-related symptoms, pain, and persistent distress related to the paralysed limb as a burdensome nonfunctional appendage. Outcomes included the Disabilities of the Arm, Shoulder and Hand (DASH) score, Visual Analog Scale (VAS) for pain, and SF-36 Physical Functioning and Emotional Well-Being domains. Results are reported descriptively as individual values and medians without hypothesis testing. **Results**: All five patients completed follow-up, which reached 30 months. After combined transhumeral amputation, biceps sling construction, RPNI, rehabilitation, and prosthetic fitting, patient-reported scores changed in this small uncontrolled series as follows. The median DASH score changed from 64 preoperatively to 50 postoperatively; in this context, DASH reflects global perceived disability and may be influenced by pain relief, altered expectations, prosthetic compensation, psychosocial adaptation, and the removal of a burdensome limb rather than restoration of upper-limb function. Median VAS pain score changed from 7 to 0; VAS captured overall pain intensity only and did not separately measure phantom limb pain, mechanical pain, neuropathic pain, deafferentation pain, or traction-related symptoms. SF-36 Physical Functioning changed from 75 to 100, and Emotional Well-Being from 75 to 100. Four patients were fitted with a myoelectric prosthesis, and one elected to use a cosmetic prosthesis. No postoperative surgical complications, socket-related complications, prosthesis abandonment, clinical shoulder dislocation, or obvious failure of the construct were observed. Two Paralympic athletes returned to competitive sport after rehabilitation. **Conclusions**: The combined procedure was technically feasible in five highly selected patients and was not associated with observed surgical or prosthetic complications during follow-up. After combined transhumeral amputation, biceps sling construction, RPNI, rehabilitation, and prosthetic fitting, patient-reported scores improved in this small uncontrolled series. The specific contribution of the biceps sling relative to amputation itself, RPNI, prosthetic rehabilitation, and patient selection remains unknown. Larger prospective studies with objective assessment of shoulder and stump stability are needed to validate these preliminary findings and refine patient-selection criteria.

## 1. Introduction

Brachial plexus injuries (BPIs) are among the most complex and disabling conditions in trauma and peripheral nerve surgery, producing both short- and long-term disability [1,2,3]. Their annual incidence is estimated at 0.8–2.4 per 100,000 inhabitants, although rates vary with geography and trauma mechanism [1,2,3,4]. Most cases involve young men exposed to high-energy events such as road-traffic collisions, motorcycle accidents, and contact sports; BPIs can also complicate traumatic shoulder dislocation. Violent traction or direct compression disrupts the plexus and may cause anything from transient neurapraxia to complete cervical root avulsion [5,6,7].

Lesions are usually classified according to Seddon or Sunderland, which grade the depth of axonal and connective-tissue damage [1,3,7]. Prognosis depends on the lesion level and timely intervention, as delays often lead to permanent neuromuscular dysfunction [1,7]. Magnetic resonance neurography, myelography, electromyography, and nerve-conduction studies guide surgical planning and help predict spontaneous recovery [1,7]. Clinically, BPIs present with severe motor and sensory deficits of the upper limb, neuropathic pain, progressive muscle atrophy and joint instability, all of which can severely diminish quality of life and prevent return to work [5,6,7]. Loss of dynamic stabilisation by the rotator cuff and periscapular muscles predisposes to glenohumeral dislocation and progressive arthropathy, further deepening disability [8,9,10]. Despite advances in trauma care and microsurgical techniques, delayed or inadequate management still leaves many patients with profound deficits, underscoring the need for better surgical solutions [7,11].

Current repairs rely on nerve grafting, direct transfers and neurotisation, which can restore useful movement in upper-plexus lesions (C5-C6) when performed within 3–6 months [1,7,8]. Targeted muscle reinnervation and distal nerve transfers expand options, but outcomes remain variable and time-dependent [12,13,14]. Denervation of the supraspinatus, infraspinatus, deltoid, and serratus anterior abolishes shoulder abduction, flexion, and rotation, leaving the joint unstable and nonfunctional [14]. When irreversible damage persists, shoulder arthrodesis offers pain relief and a stable position, but it is invasive, restricts compensatory motion, and limits future myoelectric–prosthetic control [12,13,14]. Lower-plexus injuries (C7-T1) further hamper rehabilitation because hand and wrist paralysis curtails functional gain despite tendon transfers and reinnervation techniques [1,7]. These challenges create an urgent need for alternatives that stabilise the shoulder and preserve signals suitable for bionic prostheses [1,7,14].

The reconstructive algorithm for BPI depends critically on the level of lesion (preganglionic vs. postganglionic), the timing of injury, and the availability of donor nerves. In preganglionic avulsions, particularly when involving C5–T1, donor options are limited, and even early reconstruction yields modest functional recovery. In late-presenting or globally denervated limbs, the likelihood of achieving useful hand or elbow function declines sharply, often leaving patients with a painful, flail limb despite extensive reconstruction.

This study describes a combined surgical approach comprising transhumeral amputation, a passive biceps brachii traction sling, regenerative peripheral nerve interfaces (RPNIs) performed systematically at the time of amputation, and planned prosthetic rehabilitation in selected patients with irreversible brachial plexus injury and painful flail shoulder. Amputation alone provides inconsistent analgesia, and phantom limb pain (PLP) may persist. Contemporary strategies often combine amputation with neuromodulatory or biologic interfaces such as spinal cord stimulation, peripheral nerve stimulation, targeted muscle reinnervation (TMR), and RPNI. In this series, RPNIs were performed systematically. TMR was not indicated due to the absence of reliable donor input. The sling component was designed as a passive mechanical construct intended to resist inferior glenohumeral traction and to optimise the residual limb for prosthetic fitting while preserving channels for myoelectric control [12,13,14]; it was not designed to restore active shoulder motion or replace rotator cuff or periscapular function.

The aim of this report is to describe the technical details of this combined approach and to assess its feasibility and perioperative safety descriptively. Because the series was retrospective, uncontrolled, and involved five patients receiving a complex, multi-component intervention, no causal inference regarding the specific contribution of the biceps sling to any observed clinical changes can be drawn. The hypothesis that the biceps sling provides meaningful passive resistance to inferior glenohumeral traction and improves residual limb stability remains unproven and requires objective biomechanical and radiographic evaluation in future prospective studies.

## 2. Materials and Methods

This retrospective, uncontrolled five-case series was designed as a technical note to assess the feasibility, perioperative safety, descriptive clinical outcomes, and early prosthetic integration of modified transhumeral amputation with a passive biceps brachii traction sling. The procedure was intended to provide passive resistance to inferior glenohumeral traction and to optimise a prosthesis-ready stump, not to restore active shoulder stability or replace rotator cuff, deltoid, or periscapular function. Given the small uncontrolled cohort, the study was not designed or powered to assess comparative efficacy or superiority over conventional transhumeral amputation or other reconstructive pathways.

The study was conducted at Brixsana Private Clinic and Bolzano General Hospital and included all consecutive patients with irreversible brachial plexus palsy who underwent modified transhumeral amputation incorporating a biceps brachii traction sling between January 2022 and July 2023. According to local institutional policy, formal Ethics Committee approval was waived because the study involved retrospective analysis of anonymized clinical data, in accordance with the Declaration of Helsinki. Written informed consent was obtained from all participants, including specific consent for the acquisition and publication of clinical photographs and video recordings for scientific, educational, and conference purposes.

Eligible cases showed complete loss of upper-limb function after failed reconstructive surgery for severe brachial plexus injury, disabling pain from inferior glenohumeral subluxation, no realistic further reconstructive options, and a sustained amputation request maintained for at least twelve months after detailed counselling. Preoperative assessment included routine laboratory investigations, imaging of the upper arm and glenohumeral joint, and psychological screening to exclude uncontrolled psychiatric disorders. In addition, preoperative electromyographic (EMG) mapping was performed to identify and characterise potential detection sites in the proximal upper arm and shoulder region for future control of a myoelectric bionic prosthesis (Supplementary Video S1).

For each patient, the extent and level of the brachial plexus lesion, involved nerve roots, and preoperative biceps brachii function were documented using the Medical Research Council grading system. Detailed patient characteristics are presented in Table 2.

### Surgery Technique

The biceps brachii traction sling was designed as a passive stabilising construct to resist inferior glenohumeral translation and optimise the residual limb for prosthetic fitting. It was not intended to restore active shoulder stability or replace rotator cuff, deltoid, or periscapular function.

**Figure 1:**
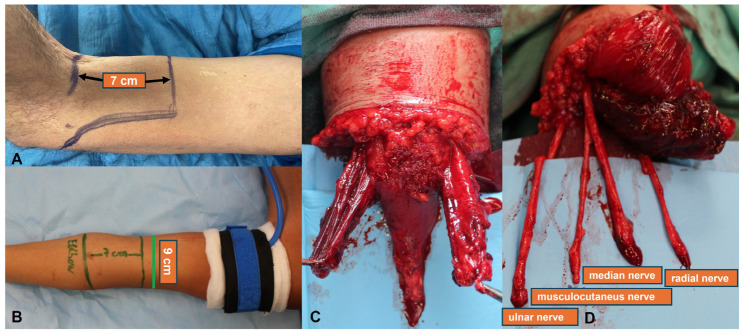
(**A**) Amputation level located 7 cm proximal to the antecubital fossa. (**B**) Supine positioning of the arm with proximal vascular control achieved using an upper-arm tourniquet. (**C**) Isolation of the brachialis and biceps brachii muscles. (**D**) Identification, isolation, and transection of the ulnar, musculocutaneous, median, and radial nerves.

The surgical procedures were performed by board-certified plastic surgeons with extensive expertise in microsurgery, peripheral nerve surgery, and bionic reconstructive surgery, assisted by junior trainees. All procedures were performed under general anaesthesia with upper-arm tourniquet control and additional axillary plexus blockade. The patient was positioned supine, and the amputation level was planned approximately 7 cm proximal to the antecubital crease (Figure 1A,B). Following skin incision and elevation of a posterior skin flap, the distal insertion of the biceps brachii tendon was identified and detached from the radial tuberosity together with a small periosteal sleeve. The brachialis muscle was then split longitudinally over approximately 5 cm, along its fibre orientation, to facilitate subsequent stump coverage and improve surgical exposure (Figure 1C). To provide additional soft tissue coverage and enhance the stability of the residual limb, the triceps brachii tendon was detached distally, and the muscle was mobilised as a muscle flap. The ulnar, musculocutaneous, median, and radial nerves were identified, mobilised, and transected (Figure 1D). The major vascular structures of the arm were ligated and divided. The humerus was circumferentially exposed over a length of approximately 9 cm, transected using an oscillating saw, and the cut surface was smoothed and rounded.

**Figure 2:**
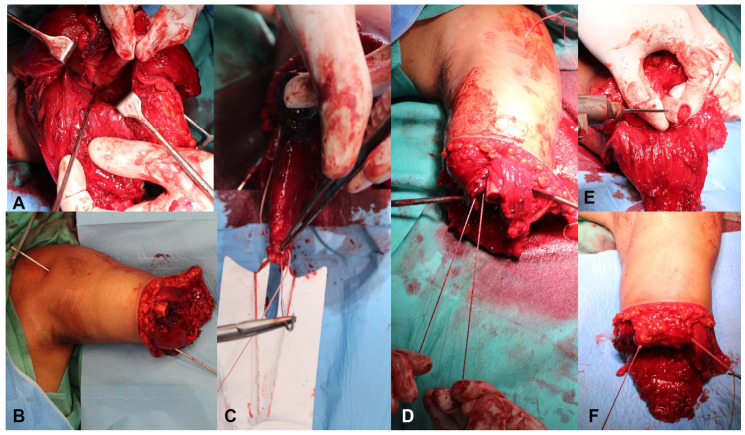
(**A**) Exposure of the humerus for approximately 8 cm. (**B**) Oblique passage of the Beath pin through the humerus. (**C**) Preparation of the detached biceps tendon for fixation. (**D**) Final biceps brachii tendon fixation in the dorsal humeral tunnel under maximal tension. (**E**) Creation of the transosseous bone tunnel. (**F**) Wrapping of the biceps brachii muscle belly around the distal humeral stump to form a muscular cap.

Passive mechanical stabilisation was achieved using a traction sling, followed by dorsal tension band fixation of the biceps brachii tendon to the humerus. For this purpose, approximately 8 cm of the humerus was exposed (Figure 2A), and the biceps brachii muscle was bluntly passed around the humerus. After determination of the optimal muscle tension by controlled traction, an eyelet guide wire was inserted obliquely from the distal dorsal aspect of the humerus and advanced proximal ventrally through the bone, exiting through the skin (Figure 2B). The guide-wire tract was subsequently enlarged with a cannulated 4.2-mm drill bit through both cortices of the humerus. Thereafter, the dorsal cortex was selectively enlarged using a cannulated 8-mm drill bit. The tendon was secured to a ToggleLoc™ with ZipLoop^®^ fixation device (13-mm ToggleLoc button with standard #7 ZipLoop) using two MaxBraid^®^ 2-0 simple interrupted sutures (Zimmer Biomet, 1800 West Center St., Warsaw, IN 46581-0587, USA) (Figure 2C). The suture limbs were shuttled through the humeral tunnel to the ventral aspect over the guide wire. Final fixation of the biceps brachii tendon was achieved within the dorsal humeral tunnel using the 13-mm ToggleLoc button while maintaining maximal tension on the musculotendinous unit (Figure 2D). At this stage, accurate repositioning of the biceps brachii muscle around the distal humeral stump is critical. To prevent displacement, the muscle was additionally secured to the distal humerus through two lateral drill holes using two MaxBraid^®^ 2-0 simple interrupted sutures (Figure 2E,F). The previously created brachialis muscle flaps, which had been split longitudinally along the direction of their muscle fibres, were subsequently wrapped around the biceps brachii muscle and apposed ventrally along the median plane using interrupted sutures.

**Figure 3:**
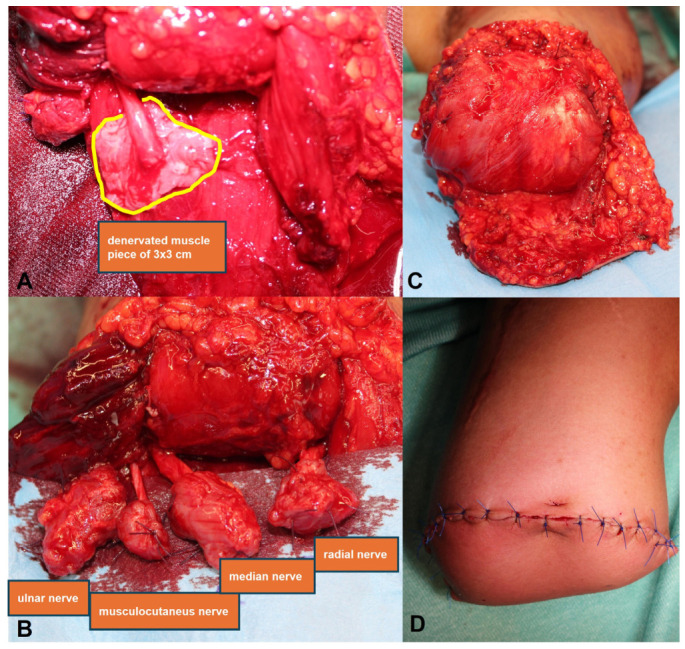
(**A**,**B**) Capping of each nerve stump and implantation into denervated muscle to create regenerative peripheral nerve interfaces. (**C**) Ventral repositioning of the triceps. (**D**) Layered closure.

For neuroma prophylaxis, the ulnar, musculocutaneous, median, and radial nerve stumps were each capped and implanted into denervated muscle targets to create RPNIs (Figure 3A,B). This was intended to reduce the risk of symptomatic neuroma-related pain and preserve the potential for future interface-based prosthetic control. The triceps brachii muscle flap was then transposed ventrally to augment soft tissue coverage and optimise contouring of the residual limb (Figure 3C). Following meticulous haemostasis, a closed-suction drain was placed. Definitive stump closure was completed with inset of the posterior skin flap and tension-free layered wound closure (Figure 3D). TMR was not performed because no reliable donor motor input was available. Spinal cord stimulation and peripheral nerve stimulation were not part of the treatment protocol.

Follow-up data were available for all patients, with a maximum follow-up of 30 months. Patient-reported outcomes (DASH, VAS, SF-36) were recorded preoperatively and at the most recent follow-up visit, which occurred no earlier than 6 months postoperatively, with final outcome evaluation completed at a maximum follow-up of 30 months (Table 3). Individual follow-up durations, timing of postoperative outcome assessment, timing of prosthetic fitting, rehabilitation protocol, occupational therapy duration, prosthesis use at last follow-up, revision surgery status, and complications per patient are reported in Table 4. Postoperative rehabilitation followed a three-phase protocol: (1) wound healing and stump maturation (weeks 0–8), supervised by the treating plastic surgeon; (2) prosthetic fitting, socket fabrication, and myoelectric control training (months 3–6) (Supplementary Video S2), supervised by a certified prosthetist and occupational therapist; and (3) functional and occupational integration (months 6–30), with structured occupational therapy sessions of 45 min twice weekly for a minimum of 10–12 weeks. Patients fitted with a myoelectric prosthesis underwent EMG-guided biofeedback training before definitive socket fitting. Rehabilitation content, prosthesis type, and daily wear duration at last follow-up are reported individually in Table 4.

No objective measures of shoulder position, glenohumeral subluxation grade, stump stability, socket load distribution, radiographic alignment, or motion analysis were obtained in this series. The claim that the biceps sling provides passive resistance to inferior glenohumeral traction, therefore, remains a design hypothesis based on anatomical and biomechanical rationale rather than a demonstrated finding. The Supplementary Videos (S3 and S4) are provided as illustrative material to document the clinical appearance and prosthetic function achieved; they do not constitute objective evidence of sling-mediated shoulder or stump stabilisation and should not be interpreted as such.

## 3. Results

**Figure 4:**
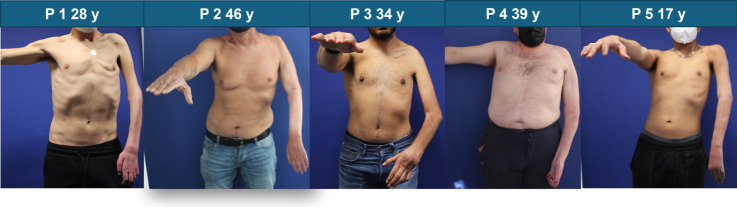
Clinical photographs of patients 1–5 (from left to right), corresponding to the demographic data presented in Table 1.

The cohort included five male patients aged 17 to 46 years, all with injuries involving the same upper extremity. Demographic and injury-related data are reported in Table 1 and Figure 4.

All patients had severe brachial plexus injury with complete loss of useful upper-limb function after previous unsuccessful reconstructive procedures. Detailed lesion characteristics, preoperative biceps function, previous treatments, main preoperative complaints, pain management, indication for transhumeral amputation, injury year, date of surgery, and injury-to-amputation interval are reported in Table 2. All patients who reported pain and distress related it to the perception of the flail limb as a nonfunctional appendage. Four of the five patients had complete preganglionic brachial plexus injury with no residual biceps function, while one patient retained partial biceps strength (MRC 3/5) following an intercostal-to-musculocutaneous nerve transfer.

Preoperative and postoperative patient-reported outcomes are summarised in Table 3. After combined transhumeral amputation, biceps sling construction, RPNI, rehabilitation, and prosthetic fitting, patient-reported scores changed as follows in this small uncontrolled series. These changes cannot be attributed to the biceps sling specifically; they may reflect amputation itself, removal of the painful and burdensome flail limb, RPNI, rehabilitation, prosthetic fitting, psychological adaptation, regression to the mean, or a combination of these factors. Median DASH score changed from 64 (IQR, 62–67) preoperatively to 50 (IQR, 47–53) postoperatively. In this setting, DASH score changes should not be interpreted as evidence of restored upper-limb function. DASH improvement in this cohort may reflect pain relief, removal of a burdensome limb, altered functional expectations, prosthetic compensation, and psychosocial adaptation rather than any improvement in native limb performance. Median VAS pain score changed from 7 (IQR, 6–7) to 0 (IQR, 0–1). VAS captured overall pain intensity only and did not separately measure phantom limb pain, mechanical traction-related pain, neuropathic pain, or deafferentation pain. The observed change in VAS, therefore, cannot be interpreted as evidence of improvement in any specific pain subtype, including phantom limb pain or traction-related pain. SF-36 Physical Functioning changed from 75 (IQR, 75–80) to 100, and SF-36 Emotional Well-Being changed from 75 (IQR, 75–80) to 100 (IQR, 95–100). These data are reported descriptively and without hypothesis testing.

All five patients proceeded to prosthetic evaluation within 3–6 months postoperatively. Four patients were fitted with a myoelectric prosthesis and reported daily wear times of 4–8 h at 30 months of follow-up. One patient elected to use a cosmetic prosthesis only because of limited occupational need. No postoperative surgical complications, socket-related complications, prosthesis abandonment, or clinical failure of the construct were observed during follow-up. Individual patient-level outcomes, including follow-up duration, timing of postoperative outcome assessment, time to prosthetic fitting, prosthesis type at last follow-up, daily wear duration, occupational therapy duration, revision surgery status, and complications, are summarised in Table 4.

**Figure 5:**
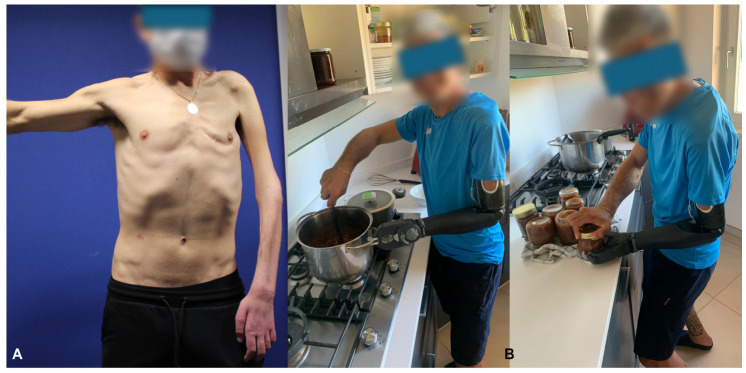
Representative preoperative and postoperative functional images. (**A**) Preoperative appearance of a painful nonfunctional flail upper limb in a patient with irreversible brachial plexus injury. (**B**) Postoperative prosthetic integration during an activity of daily living after transhumeral amputation with biceps brachii traction sling, prosthetic fitting, and rehabilitation.

Representative preoperative appearance and postoperative functional use of the prosthesis in patient 1 are shown in Figure 5. The Supplementary Videos S3 and S4 further demonstrate the functionality of the prosthesis and the stability of the shoulder joint during a training exercise performed by patient 1; these videos are illustrative only and do not constitute objective evidence of sling-mediated stabilisation. No objective radiographic, biomechanical, or socket-load measurements of shoulder position, glenohumeral subluxation, or stump stability were obtained preoperatively or postoperatively. The absence of clinical dislocation and socket-related complications during follow-up is a descriptive safety observation only and cannot be interpreted as evidence that the sling provided effective passive glenohumeral stabilisation.

Of note, two patients (P1 and P3) were Paralympic athletes and returned to competitive sport after amputation, prosthetic fitting, and rehabilitation, subsequently achieving competitive success in multiple events. Given the small sample size and descriptive design, no inferential statistical analysis was performed. Outcomes are therefore presented descriptively at the individual-patient level.

## 4. Discussion

This retrospective five-case series provides preliminary data on a combined surgical approach, comprising elective transhumeral amputation, a passive biceps brachii traction sling, RPNI, and planned prosthetic rehabilitation. In this highly selected cohort, all patients showed improvement in pain, patient-reported disability, and selected SF-36 domains. Notably, all patients reported preoperative overall pain, comprising PLP, mechanical traction-related pain, neuropathic pain, deafferentation pain, and distress associated with perceiving the flail limb as a nonfunctional and burdensome appendage. In this context, the observed improvements should be interpreted not merely as changes in questionnaire scores, but also as clinically meaningful relief from the physical and psychological burden of a painful, insensate, and nonfunctional upper limb [1,7,14]. These benefits were maintained during the available follow-up period, which extended up to 30 months, with no clinical dislocation, socket-related complication, prosthesis abandonment, or obvious failure of the construct observed. Rather, they likely reflect the combined effects of the complex intervention, including amputation itself, removal of the painful and burdensome flail limb, RPNI, rehabilitation, prosthetic fitting, psychological adaptation, regression to the mean, and other contextual factors inherent to this treatment pathway.

These benefits were maintained during the available follow-up period, which extended up to 30 months, with no clinical dislocation, socket-related complications, prosthesis abandonment, or obvious failure of the construct observed. However, these changes cannot be attributed to the biceps sling specifically. Rather, they likely reflect the combined effects of the complex intervention, including amputation itself, removal of the painful and burdensome flail limb, RPNI, rehabilitation, prosthetic fitting, psychological adaptation, regression to the mean, and other contextual factors inherent to this treatment pathway.

The main contribution of this report is not the general concept of myoplasty or myodesis, which is well established in transhumeral amputation surgery [11]. Rather, it describes a structured biceps brachii-based passive traction sling applied to the specific and challenging setting of flail shoulder after irreversible BPI. In these patients, loss of active glenohumeral and periscapular control exposes the shoulder and residual limb to inferior traction, limb encumbrance, mechanical discomfort, and difficulty with prosthetic fitting [1,7,14]. The described construct was designed to redirect the residual biceps muscle-tendon unit around the distal humeral stump, creating a passive suspension vector intended to improve stump contour, reduce inferior traction, and facilitate prosthetic rehabilitation. Therefore, the technique should be interpreted as a modified passive myodesis/sling configuration rather than as a dynamic stabilising procedure or as a proven alternative superior to conventional transhumeral amputation techniques [11,14].

The biomechanical rationale of the sling remains hypothetical. The central hypothesis is that wrapping the biceps muscle-tendon unit around the distal humeral stump creates a passive suspension vector that resists inferior glenohumeral traction and improves residual limb contour and prosthetic socket stability in patients with total or near-total brachial plexus palsy. In the present cohort, four of five patients had complete preganglionic brachial plexus injury with absent biceps function, and one patient retained partial biceps strength after previous intercostal-to-musculocutaneous nerve transfer. Because the biceps was denervated in the majority of cases, the sling was not expected to provide active shoulder motion; it was intended as a passive structural suspension. Denervated muscle undergoes progressive atrophy and cannot provide dynamic glenohumeral control. No objective measures of shoulder position, subluxation grade, stump stability, socket load, radiographic alignment, or biomechanical performance were obtained. The Supplementary Videos are illustrative clinical documentation only and do not constitute evidence of sling efficacy. The absence of clinical dislocation and socket-related complications during follow-up is a descriptive safety observation that is consistent with, but does not confirm, the hypothesised mechanism.

Pain reduction was among the most relevant clinical findings. Median VAS pain score changed from 7 to 0, suggesting substantial relief of the symptoms associated with the painful flail limb. However, VAS captured overall pain and did not distinguish between mechanical pain, neuropathic pain, deafferentation pain, and PLP. These pain components are biologically and clinically distinct, and amputation does not necessarily address centrally mediated neuropathic pain [1,7]. Because PLP, traction-related pain, mechanical pain, and neuropathic pain were not measured separately using validated instruments, the observed VAS change cannot be interpreted as evidence of improvement in any of these specific pain subtypes. The observed change in overall VAS may partly reflect the removal of the weight and encumbrance of the flail limb, reduction in traction-related symptoms, improved residual limb stability, rehabilitation, and prosthetic fitting. Therefore, the observed improvement should not be interpreted as direct evidence that the biceps sling specifically reduced neuropathic pain or PLP. Rather, it supports the clinical relevance of a combined strategy addressing mechanical instability, neuroma prophylaxis, and prosthetic readiness [1,7,14].

RPNIs were systematically performed in this cohort as part of the reconstructive strategy. This was intended to reduce the risk of symptomatic neuroma-related pain and to preserve the possibility of future interface-based prosthetic control strategies [3,14]. In contrast, TMR was not performed because no reliable donor motor input was available. TMR requires dependable proximal motor signals, and the predominance of pan-plexus avulsion without residual biceps function made this approach unsuitable in the present series. Neuromodulatory approaches such as spinal cord stimulation or peripheral nerve stimulation may be considered in selected refractory cases and may act synergistically with biologic interfaces, although these modalities were not used in the present cohort [1,7,14].

The improvement in SF-36 Physical Functioning and Emotional Well-Being suggests that the procedure may have effects beyond pain reduction; however, these changes occurred in the context of a complex, multi-component intervention and cannot be attributed to the sling specifically. In total BPI, a flail and nonfunctional limb may contribute to distress, altered body image, social limitation, and reduced autonomy [1,7,14]. In this setting, removal of the painful and insensate limb, reduction in limb weight, and creation of a prosthesis-ready stump may support psychosocial adaptation and improve perceived quality of life [7,8,9,10,11,14]. Similarly, DASH score improvement in this setting should not be interpreted as evidence of restored upper-limb function. In patients with a previously nonfunctional and burdensome flail limb, DASH changes may primarily reflect pain relief, removal of the limb’s encumbrance, altered functional expectations following amputation, prosthetic compensation, and psychosocial adaptation rather than any change in native upper-limb performance.

Four patients were fitted with myoelectric prostheses and achieved daily use, while one patient elected to use a cosmetic prosthesis because of limited occupational need. Of particular clinical relevance, two patients who were Paralympic athletes returned to competitive sport after surgery, prosthetic fitting, and rehabilitation, subsequently achieving important competitive results. This observation highlights the potential impact of the procedure not only on pain and prosthetic readiness, but also on social reintegration, participation, and patient-perceived autonomy.

Elective amputation for irreversible BPI remains uncommon in Europe and is generally reserved for carefully selected patients after failed reconstructive attempts [2,7,11]. In contrast, several Asian centres, particularly in Japan, have traditionally prioritised limb-preserving strategies such as nerve transfers and free functional muscle transplantation [15,16]. These approaches can achieve partial functional restoration and social reintegration without amputation, but they often require multiple staged procedures and prolonged rehabilitation and may provide variable return of meaningful function [17]. The present series reflects a complementary strategy in which, for selected patients with non-reconstructable lesions, persistent disability, and a sustained request for amputation after counselling, TA combined with passive stump stabilisation, RPNI-based nerve management, and planned prosthetic rehabilitation may represent a realistic reconstructive endpoint rather than a failure of treatment.

The decision to proceed with elective amputation should remain cautious and multidisciplinary, involving surgeons, physiatrists, occupational therapists, psychologists, pain specialists, and the patient [1,7,14]. Preoperative counselling is essential to clarify realistic functional expectations, address the possibility of persistent neuropathic or PLP, and reduce the risk of prosthesis rejection, which remains a relevant issue in upper-limb amputation [1,7,14]. The present findings may be of interest to centres treating complex irreversible brachial plexus injuries, particularly when conventional nerve reconstruction, tendon transfer, free functional muscle transplantation, or arthrodesis are unlikely to provide acceptable functional recovery or symptom control.

Several limitations must be acknowledged. This was a retrospective uncontrolled series of five patients, and the findings should be considered preliminary and hypothesis-generating. First, the multi-component nature of the intervention, comprising elective amputation, biceps sling construction, RPNI, rehabilitation, and prosthetic fitting, means that the specific contribution of any single element, including the sling, cannot be isolated. The observed changes in DASH, VAS, and SF-36 may reflect amputation itself, removal of a painful and burdensome limb, RPNI, rehabilitation, prosthetic fitting, psychological adaptation, regression to the mean, or other contextual factors, rather than any specific effect of the biceps sling. Second, no objective biomechanical, radiographic, socket-load, motion-analysis, or EMG-based assessment of shoulder position, glenohumeral subluxation grade, or stump stability was performed preoperatively or postoperatively. The mechanistic claim that the sling provides passive resistance to inferior glenohumeral traction, therefore, remains an unproven design hypothesis that requires objective investigation. Third, pain was assessed using VAS, which did not distinguish between mechanical pain, neuropathic pain, deafferentation pain, traction-related pain, and PLP. PLP and traction-related pain were not measured with validated instruments, and the VAS change cannot be attributed to improvement in any specific pain subtype. Fourth, DASH should be interpreted as a measure of global perceived disability rather than proof of restored native upper-limb function. In this cohort, DASH changes may primarily reflect psychosocial adaptation, altered expectations, and removal of a burdensome limb rather than functional improvement. Fifth, no comparison with conventional transhumeral amputation without a sling was made, and no control group was available. Future prospective multicentre studies with larger cohorts, standardised rehabilitation protocols, objective assessment of glenohumeral and stump stability with radiographic and biomechanical methods, control groups, longer follow-up, and validated neuropathic and PLP instruments will be necessary to confirm these preliminary observations and refine patient-selection criteria.

## 5. Conclusions

This study describes a technical modification of transhumeral amputation incorporating a passive biceps brachii-based sling configuration for selected patients with irreversible brachial plexus injury and painful flail shoulder. In this five-case series, the combined procedure was technically feasible in five carefully selected patients and was not associated with observed surgical or prosthetic complications during the available follow-up. After combined transhumeral amputation, biceps sling construction, RPNI, rehabilitation, and prosthetic fitting, patient-reported scores improved in this small uncontrolled series. The specific contribution of the biceps sling to these observations, relative to amputation itself, RPNI, prosthetic rehabilitation, and patient selection, remains unknown and cannot be determined from this study design. The hypothesis that the sling provides passive resistance to inferior glenohumeral traction requires objective biomechanical and radiographic confirmation. Prospective multicentre studies with larger cohorts, standardised rehabilitation protocols, control groups, and objective assessment of stump and shoulder stability are needed to determine the specific contribution of the sling component and to define its potential role within the reconstructive algorithm for irreversible brachial plexus injury.

## Figures and Tables

**Table 1:** Patient demographics and clinical characteristics at the time of transhumeral amputation.

Subject	Age	Sex	Affected Limb	Paralysis Type
1	28	M	Left	Mixed, predominantly preganglionic
2	46	M	Left	Preganglionic complete
3	34	M	Left	Preganglionic complete
4	39	M	Left	Preganglionic complete
5	17	M	Left	Preganglionic complete

**Table 2:** Clinical characteristics, preoperative complaints, and timing of TA. BPI = Brachial Plexus Injury; TA = Transhumeral Amputation.

Patient	Injury Year	TA Date	Brachial Plexus Lesion	Biceps Function	Previous Treatment	Main Preoperative Complaints and Pain Management	Main Indication for TA	Injury to TA Interval
1	2016	9 December 2022	Mixed left BPI: partial C5 avulsion and complete C6-T1 avulsion, with traumatic pseudomeningoceles and denervation changes in shoulder girdle muscles	MRC 3/5 after intercostal-to-musculocutaneous nerve transfer	Multiple unsuccessful reconstructive procedures	Pain and distress related to a nonfunctional flail limb; pregabalin and duloxetine were discontinued after TA with temporary benefit.	Sublucation, trauma risk, and limb encumbrance	~6 years
2	2012	1 February 2023	Total left BPI	Absent	Failed nerve transfers	Pain and distress related to a nonfunctional flail limb; partial control with pregabalin/amitriptyline.	Subluxation and limb encumbrance	~11 years
3	2016	26 April 2023	Complete left BPI with associated vascular and skeletal injury	Absent; compensatory movement through trapezius	Revascularization and nerve reconstruction	Pain and distress related to a nonfunctional flail limb; chronic neuropathic pain without continuous pharmacological treatment.	Sublucation, trauma risk, and limb encumbrance	~7 years
4	2012	17 May 2023	Total left BPI	Absent	Failed reconstructive attempts	Pain and distress related to a nonfunctional flail limb; limited response to duloxetine/low-dose opioids	Subluxation, trauma risk, and prosthetic use	~11 years
5	2020	31 July 2023	Total left BPI	Absent	Failed reinnervation attempts	Pain and distress related to a nonfunctional flail limb; limited response to gabapentin	Subluxation, trauma risk, and prosthetic use	~3 years

**Table 3:** Summary of preoperative and postoperative patient-reported outcomes up to 30 months. DASH = Disabilities of the Arm, Shoulder and Hand; VAS = Visual Analog Scale; SF-36 = Short Form-36; PF = Physical Functioning; EW = Emotional Well-Being; IQR = interquartile range.

Patient	DASH Pre	DASH Post	VAS Pre	VAS Post	SF-36 PF Pre	SF-36 PF Post	SF-36 EW Pre	SF-36 EW Post
1	67	47	6	0	80	100	80	100
2	64	50	7	0	70	100	75	100
3	60	53	7	0	75	100	75	100
4	62	44	6	0	85	100	80	100
5	71	59	7	1	75	100	65	95
Median (IQR)	64 (62–67)	50 (47–53)	7 (6–7)	0 (0–1)	75 (75–80)	100	75 (75–80)	100 (95–100)

**Table 4:** Patient-level postoperative outcomes. OT = occupational therapy.

Patient	Follow-Up Duration (Months)	Timing of Outcome Assessment (Months)	Time to Prosthetic Fitting (Months)	Prosthesis Type at Last Follow-Up	Daily Wear Duration at Last Follow-Up	OT Duration (Weeks)	Revision	Complications
1	30	6 and 30	3	Myoelectric	4–8 h	12	None	None
2	30	6 and 30	4	Myoelectric	4–6 h	12	None	None
3	30	6 and 30	3	Myoelectric	4–6 h	12	None	None
4	30	6 and 30	4	Myoelectric	6–8 h	10	None	None
5	30	6 and 30	6	Cosmetic only	4–8 h	12	None	None

## Data Availability

All relevant data included in the study are given in this manuscript and are available from the corresponding author on reasonable request (alexander.gardetto@brixsana.it).

## Supplementary Materials

The following supporting information can be downloaded at https://www.mdpi.com/article/10.3390/jcm15135284/s1. Video S1 (EMG Mapping), Video S2 (early postoperative training of myoelectric prosthetic control), Video S3 (demonstration of the functionality of the prosthesis), and Video S4 (stability of the shoulder joint) are included.

## Abbreviations

The following abbreviations are used in this manuscript:

BPI	Brachial Plexus Injury
TA	Transhumeral Amputation
RPNI	Regenerative Peripheral Nerve Interface
TMR	Targeted Muscle Reinnervation
EMG	Electromyography
DASH	Disabilities of the Arm, Shoulder, and Hand
VAS	Visual Analog Scale
PF	Physical Functioning
EW	Emotional Well-Being
IQR	Interquartile Range

## References

[1] Pejkova S., Filipce V., Peev I., Nikolovska B., Kalpakci A., Stankov O., Stojanoski V. (2021). Brachial plexus injuries—Review of the anatomy and the treatment options. Prilozi.

[2] Lee E.Y., Pulos N., Bishop A.T., Shin A.Y. (2024). The failed adult traumatic brachial plexus reconstruction. J. Hand Surg. Eur. Vol..

[3] Mioton L.M., Dumanian G.A. (2018). Targeted muscle reinnervation and prosthetic rehabilitation after limb loss. J. Surg. Oncol..

[4] Figueroa B.A., Ordenana C.X., Rezaei M., Said S.A., Dugan A.J., Shores J.T., Gastman B.R. (2024). Orthotopic forelimb transplantation in a Yucatan minipig model: Anatomic and in vivo study. Microsurgery.

[5] Steinberger Z., Xu H., Kazmers N.H., Li Z., Yao J. (2017). Elbow vascularized composite allotransplantation—Surgical anatomy and technique. J. Shoulder Elb. Surg..

[6] Rudzki J.R., Shaffer B.S., Cole B.J., Sekiya J.K. (2015). Arthroscopic treatment of biceps tendinopathy. Operative Techniques in Shoulder and Elbow Surgery.

[7] Bahm J. (2023). Invited Contributions: Fields Outside of My Work. Surgical Rationales in Functional Reconstructive Surgery of the Upper Extremity.

[8] Rodriguez M.C., Shin A.Y. (2023). Adult brachial plexus injuries: Treatment in the acute phase. Cirugía De Mano Y Microcirugía.

[9] Haas F., Hubmer M., Koch H., Parvizi D., Scharnagl E., Pierer G. (2004). Immediate functional transfer of the latissimus dorsi myocutaneous island flap for reestablishment of elbow flexion in upper arm replantation: Two clinical cases. J. Trauma Acute Care Surg..

[10] Albo E. (2024). The Agonist-Antagonist Myoneural Interface (AMI) in the Surgical Revision of Upper Limb Amputation for the Proprioceptive Feedback and Advanced Control of Bionic Polyarticulated Prosthesis. Ph.D. Thesis.

[11] Larsen C.G., Griffis M., Tanner N., Tedesco L.J., Barfield W.R., Wheeless C.R. (2023). Optimizing transhumeral amputations. Oper. Tech. Orthop..

[12] Dabestani P.J., Ramsey M.D., Chappell A.G., Hoelzer B., Handorf C., Milbrandt T.A. (2022). Free vascularized fibular flap with bilateral bipolar latissimus transfer for upper extremity reconstruction: A case report. JBJS Case Connect..

[13] Novo J., Seth I., Mon Y., Soni A., Elkington O., Marcaccini G., Rozen W.M. (2025). Use of Robotic Surgery in Plastic and Reconstructive Surgery: A Narrative Review. Biomimetics.

[14] Nanos G.P., Dromsky D., McKay P.L., Owens B.D. (2014). Arm and shoulder injuries. Fundamentals of Combat-Related Extremity and Orthopaedic Surgery.

[15] Terzis J.K., Kostopoulos V.K. (2010). Free muscle transfer in posttraumatic plexopathies: Part 1: The shoulder. Ann. Plast. Surg..

[16] Nagano A. (1998). Treatment of brachial plexus injury. J. Orthop. Sci..

[17] Satbhai N.G., Doi K., Hattori Y., Sakamoto S. (2016). Functional outcome and quality of life after traumatic total brachial plexus injury treated by nerve transfer or single/double free muscle transfers. Bone Jt. J..

